# Apneustic anesthesia ventilation improves pulmonary function in anesthetized bottlenose dolphins (*Tursiops truncatus*)

**DOI:** 10.3389/fvets.2024.1287478

**Published:** 2024-04-05

**Authors:** Carolina R. Le-Bert, Alex Bukoski, John Downs, David S. Hodgson, Lori Thombs, Sam H. Ridgway, James Bailey

**Affiliations:** ^1^U.S. Navy Marine Mammal Program, Naval Information Warfare Center Pacific, San Diego, CA, United States; ^2^Department of Veterinary Medicine and Surgery, College of Veterinary Medicine, University of Missouri, Columbia, MO, United States; ^3^Department of Anesthesiology, College of Medicine, University of Florida, Gainesville, FL, United States; ^4^Innovative Veterinary Medicine, Ponte Vedra, FL, United States; ^5^Department of Clinical Sciences, College of Veterinary Medicine, Kansas State University, Manhattan, KS, United States; ^6^Department of Statistics, College of Arts and Science, University of Missouri, Columbia, MO, United States; ^7^U.S. Navy Marine Mammal Program, National Marine Mammal Foundation, San Diego, CA, United States

**Keywords:** bottlenose dolphin, mechanical ventilation, apneustic anesthesia ventilation, pulmonary physiology, anesthesia, physical status classification

## Abstract

**Introduction:**

Use of mechanical ventilation during general anesthesia is a necessary practice in the anesthetization of small cetaceans as spontaneous ventilation fails to provide adequate gas exchange. Currently available methods of ventilation do not account for the intermittent breathing strategy of representative species within this infraorder of fully aquatic mammals and may have a significant effect on cardiac and respiratory physiology.

**Methods:**

To understand the impact of mechanical ventilation on cardiopulmonary function in one small species of cetacean, the bottlenose dolphin (*Tursiops truncatus*), we compared controlled mechanical ventilation (CMV) to a novel ventilation method known as apneustic anesthesia ventilation (AAV). AAV simulates the normal inspiratory breath-hold pattern of dolphins. Ten anesthetic procedures (dental procedure, *n* = 9; bronchoscopy, *n* = 2) were performed on nine dolphins (age range: 10–42 years; mean = 32 years; median = 37 years; female = 3, 40%; male = 6, 60%). In a cross-over study design, dolphins were instrumented and randomly assigned to AAV or CMV as the initial mode of ventilation, then switched to the alternate mode. Baseline cardiopulmonary data were collected and again after 30 min on each mode of ventilation. Cardiac index, stroke volume index, systemic vascular resistance, alveolar dead space, alveolar-arterial oxygen tension gradient, arterial oxygen content, oxygen delivery index, and dynamic respiratory system compliance index were calculated at each of the four time points.

**Results:**

During AAV, dolphins had higher arterial oxygen tension, higher mean airway pressure, reduced alveolar dead space ventilation and lower alveolar-arterial oxygen difference. Cardiovascular performance was not statistically different between the two modes.

**Discussion:**

Our study suggests AAV, which more closely resembles the conscious intermittent respiratory pattern phenotype of dolphins, improves ventilation and pulmonary function in the anesthetized dolphin. Future studies should evaluate the cardiopulmonary effects of neutral buoyancy and cardiopulmonary sparing drug protocols to reduce the need for hemodynamic support of current protocols.

## 1 Introduction

Within the infraorder of fully aquatic mammals, *Cetacea*, the bottlenose dolphin (*Tursiops truncatus*) has been well represented under the care of humans for over 100 years (1). Despite the large number of dolphins under human care, relatively few have been safely anesthetized for clinical procedures and recovered (2–5). The current limited body of literature on cetacean anesthesia focuses on drug combinations and techniques for rapid induction of anesthesia but does not address the physiologic effects of these approaches (4–12). Specifically, no literature exists on the influences of current ventilation protocols on cetacean cardiopulmonary physiology. This data gap is largely due to the paucity of controlled physiologic studies and the stagnated development of advanced methods for supportive anesthetic care in dolphins. These critical factors have been a remarkable hurdle in furthering our knowledge, understanding, and optimization of dolphin anesthesia.

As a completely aquatic marine mammal, the bottlenose dolphin evolved specialized cardiovascular and pulmonary features to facilitate breath-hold diving and underwater foraging activities (13–15). Such adaptations include, but are not limited to, periarterial venous rete for efficient thermoregulation, valve-less peripheral veins, rete mirabilia surrounding central nervous system structures, an intermittent, breath-holding breathing pattern, and reinforcement of pulmonary bronchioles with cartilage (16–20). These cardiopulmonary adaptations should be considered when performing general anesthesia, whereby ventilation and pharmacologic strategies may modulate gas-exchange mechanisms, autonomic nervous system function and responses, and vital organ perfusion states.

In our clinical experience, similar to large terrestrial mammals, cardiopulmonary derangements such as hypoventilation, ventilation-perfusion mismatch, vasodilation, and depression of cardiac contractility likely occur in anesthetized dolphins when applying commonly accepted anesthetic drugs (e.g., opioids, propofol, benzodiazepines, and inhalation anesthetics) and conventional ventilation strategies (2). It would then follow that these effects may lead to arterial hypoxemia, hypercapnia, hypotension, and decreased cardiac output. For example, respiratory depression is a characteristic of dolphin sedation and anesthesia that demands rapid intubation and respiratory support in the form of mechanical ventilation. The use of propofol and inhalation anesthetics are known to contribute to profound vasodilation and hypotension in various species and, thus, may impair circulation in the dolphin (21, 22). If not properly mitigated, these derangements can affect organ perfusion, reduce oxygen delivery, and predispose the animal to organ injury (e.g., acute kidney injury, myocardial injury, hypoxic brain injury), and may even contribute to neuropathic and myopathic conditions (23, 24). In large, completely aquatic mammals, like the dolphin, little is known about the pathophysiologic influences of anesthesia on respiratory gas exchange efficiency and organ perfusion. Thus, there is a need to understand the physiologic impacts of anesthesia in a species with unique and specialized structural and physiologic adaptations, as well as to develop strategies to alleviate and reduce anesthesia-associated physiologic derangements.

Controlled mechanical ventilation (CMV) with supplemental oxygen is a well-defined strategy used for minimizing the impact of anesthesia-induced respiratory abnormalities (e.g., hypoventilation, hypercapnia, and hypoxemia) in large aquatic mammals (2, 4, 5, 9, 25–30). While various approaches to mechanical ventilation are used within the field of human anesthesiology, available large animal veterinary anesthesia equipment has limited options. Herein, we define CMV (also referred to as conventional mechanical ventilation) as intermittent positive pressure ventilation in the absence of positive end-expiratory pressure and alveolar recruitment maneuvers (31). As defined by the authors, CMV most closely resembles the normal respiratory pattern of most non-hibernating terrestrial mammals. The dolphin, on the contrary, has an intermittent, inspiratory breath-hold respiratory pattern with significant heart rate variation during each inspiratory-to-expiratory cycle, also referred to in clinical medicine as respiratory sinus arrhythmia (32–36). While this cardiopulmonary coupling strategy may improve gas exchange in the conscious diving dolphin, the heart rate variation associated with intermittent breathing converts to a normal sinus rhythm following induction of anesthesia and initiation of mechanical ventilation (33, 37). In addition, dolphin alveoli readily collapse, pushing gases into the proximal reinforced terminal bronchioles as a proposed strategy to reduce gas exchange and oxygen consumption when diving (38). Mechanical ventilation with 100% oxygen can also promote alveolar collapse over time via absorption atelectasis (39–41). Thus, the dolphin may be more likely to experience atelectasis from alveolar collapse when anesthetized with high fractions of inspired oxygen (FIO_2_) and ventilated using traditional CMV paradigms. For these reasons, we sought to evaluate a novel mechanical ventilation approach, known as apneustic anesthesia ventilation (AAV), that may address physiologically appropriate ventilation needs of the dolphin, thereby supporting optimal cardiopulmonary function (31, 42). This mechanical ventilation strategy more closely resembles the intermittent respiratory pattern phenotype of the dolphin and therefore is worthy of investigation in the target population (26, 30, 43).

**Figure 1:**
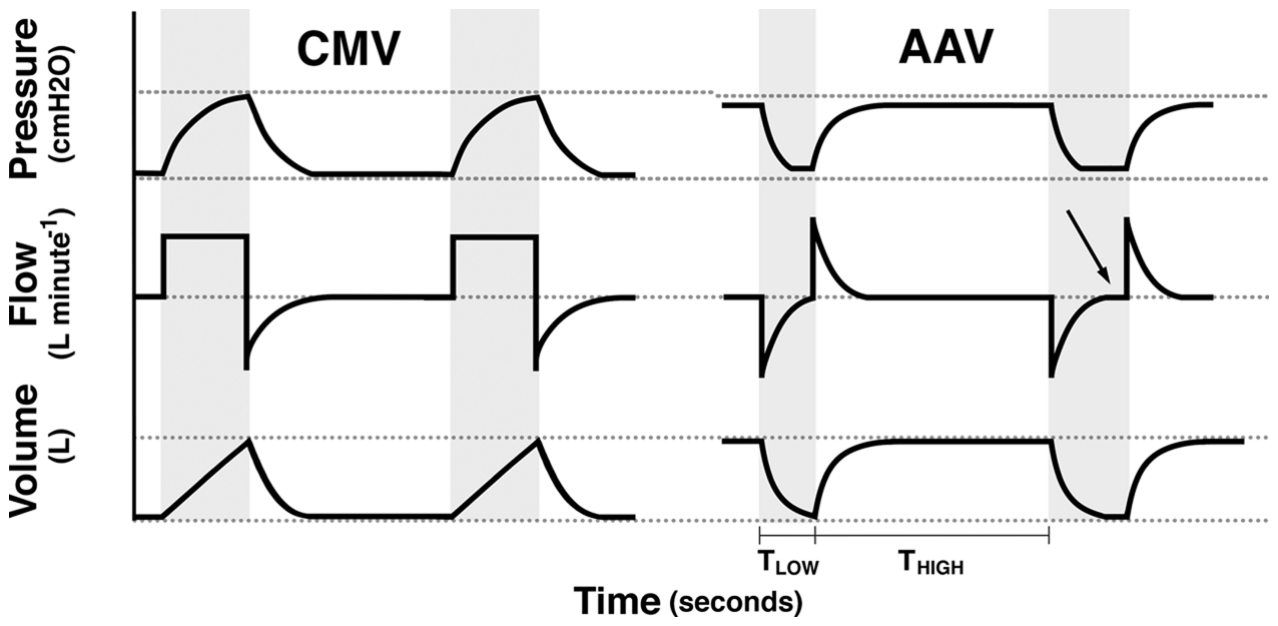
Schematic illustration of the pressure, flow, and volume vs. time waveforms for controlled mechanical ventilation (CMV) and apneustic anesthesia ventilation (AAV). Gray background indicates periods of tidal ventilation corresponding to inspiratory time during CMV and the time (T_LOW_) at P_LOW_ during AAV. White background indicates expiratory time during CMV and the time (T_HIGH_) of P_HIGH_ during AAV. For AAV waveforms, the first release, illustrates proper limitation of release time whereas the second release illustrates a long release time with flow stagnation (arrow). Reproduced with permission from Innovative Veterinary Medicine, Inc.

CMV increases airway pressure from ambient to higher pressures in order to produce tidal ventilation. In doing so, lung volume at the start of inspiration is typically below functional residual capacity (FRC; e.g., the lung volume below which alveoli are prone to collapse). In contrast, AAV maintains airway pressure above ambient, and therefore above FRC and produces tidal ventilation by decreasing pressure from this continuously applied, elevated baseline pressure (P_high_) to a lower pressure (P_low_) with the goal of maintaining lung volume at or slightly above FRC (Figure 1) (31, 43). The time at P_high_, referred to as T_high_, is significantly longer than the time at P_low_ (T_low_). This strategy promotes maintenance of lung volume and alveolar recruitment to minimize the progressive formation of anesthesia-associated atelectasis while optimizing respiratory system compliance. In human patients, AAV was shown to result in significant reduction in dead-space ventilation, improved respiratory system compliance and a lower arterial to alveolar carbon dioxide tension difference (43). In dorsally recumbent, anesthetized horses inspiring 38%–47% O_2_, respiratory system dynamic compliance was higher and venous admixture was lower with AAV compared to CMV. However, no notable improvement in cardiovascular performance was evident in this model (31). In human patients, AAV may exhibit improved cardiovascular performance compared to CMV by augmenting venous return secondary to the use of lower mean airway pressures, while minimizing ventilation-perfusion mismatch (43).

The objective of the study described here was to compare the cardiopulmonary effects of AAV to CMV, while using a repeatable anesthetic drug combination, in anesthetized bottlenose dolphins undergoing clinically indicated medical or surgical procedures. Based on the equine ventilation investigative model and clinical experience with cetacean anesthesia, we hypothesized the use of AAV would result in improved indices of pulmonary function in dolphins when compared to a traditional implementation of CMV.

## 2 Materials and methods

### 2.1 Animals

Nine (age range: 10–42 yrs.; mean: 32 yrs.; median: 37 yrs.; 3 female, 6 male) bottlenose dolphins, housed and cared for by the U.S. Navy Marine Mammal Program (MMP), Naval Information Warfare Center (NIWC) Pacific, in San Diego Bay (CA, United States), were anesthetized in a randomized, crossover design without a washout period to compare the cardiopulmonary effects of the two mechanical ventilation modes. Ventilation mode starting order was randomly assigned using a binary random number generator (www.random.org; Ireland). One female dolphin was anesthetized on 2 separate occasions separated by approximately 1 year, for a total of 10 anesthetic procedures on 9 dolphins.

As an index of general health and a prognostic tool for anesthesia-related complications, the physical status of individual dolphins were classified using an adapted version of the American Society of Anesthesiologists (ASA) physical status classification system (Table 1) (44). All animals were anesthetized for clinically indicated procedures and received comprehensive health evaluations by a veterinarian at least 7–10 days prior to the anesthetic procedures (Table 2). Health evaluations consisted of physical examinations including weight, clinicopathologic analyses (complete blood count with differential, plasma biochemical profile, and blood gas analysis sampled from the fluke periarterial venous rete), thoracic and abdominal ultrasound examinations, and electrocardiographic tracing evaluation. Animals were either excluded from the study or the procedure was delayed, if health evaluations exhibited any of the following: clinicopathologic abnormalities for the population, abnormal electrocardiographic tracings, and/or abnormal thoracic and abdominal ultrasound findings (34, 45–48). In a previous study in horses, statistically significant differences were found with an *a priori* power analysis of variance (ANOVA) design with α = 0.05, 1 − β = 0.8, effect size of 0.5 and repeated-measures correlation of 0.5 suggesting a sample size of 10 (31). For this reason, as well as the limited availability of dolphins requiring general anesthesia to address clinical disease, 10 dolphins were included in this study.

**Table 1:** Physical status classification with definitions for adult bottlenose dolphins enrolled in this study and adapted from the American Society of Anesthesiologists physical status (ASA PS) classification score for humans.

ASA PS classification	Definition	Bottlenose dolphin examples
I	A normal healthy animal	Healthy, normal body condition, with normal lungs and no history of renal nor hepatic insufficiency, elective surgical procedure
II	An animal with mild systemic disease	Mild diseases without substantive functional limitations. Obese body condition, dental pathology, ocular pathology, mild lung disease, mild musculoskeletal disease (OA), non-clinical heart disease, well controlled dysrhythmias
III	An animal with severe systemic disease	Substantive functional limitations, such as moderate lung disease, active hepatitis, chronic renal disease with treatment, moderate reduction in ejection fraction, malnutrition, gastric foreign body, congenital neurologic or cardiac disease, regional neoplasia
IV	An animal with severe systemic disease that is a constant threat to life	Severe lung and/or heart disease, acute renal disease (severe azotemia and/or uremia from stone obstruction or nephrotoxin), shock, sepsis, DIC, localized ischemic or obstructed bowel, metastatic neoplasia
V	A moribund animal that is not expected to survive without the surgery	Massive trauma, intracranial bleed with mass effect, ischemic bowel in the face of significant cardiac pathology or multiple organ dysfunction
VI	A declared brain-dead animal whose organs are being removed for donor purposes	NA
E	Denotes emergency surgery^*^	

NA, not applicable. ^*^An emergency is defined as existing when delay in treatment would lead to significant increase in threat to life or body part (i.e., acute ocular or limb/body trauma).

**Table 2:** Signalment, American Society of Anesthesiologists (ASA) physical status classification, and clinical procedures for bottlenose dolphins, *Tursiops truncatus*, enrolled in the study.

Animal ID	Sex	Age	Weight (kgs)	ASA classification	Clinical procedure
Tt-1a^*^	F^*^	37	187	II	Dental procedure
Tt-2	M	29	191	II	Dental procedure
Tt-3	M	33	254	II	Dental procedure
Tt-1b*	F*	38	188	II	Dental procedure
Tt-5	M	42	260	II	Dental procedure
Tt-6	F	16	141	II	Dental procedure
Tt-7a	M	40	292	II	Dental procedure; oral and skin biopsies
Tt-8	F	40	199	II	Bronchoscopy; Dental procedure
Tt-9	M	10	186	II	Bronchoscopy
Tt-10	M	37	191	II	Dental procedure

^*^Denotes same female dolphin anesthetized on two separate occasions.

Secretary of Navy Instruction 3900.41H directs that Navy marine mammals be provided the highest quality of care. NIWC Pacific is accredited by AAALAC International and adheres to the national standards of the U.S. Public Health Service policy on the Humane Care and Use of Laboratory Animals and the Animal Welfare Act. NIWC Pacific’s animal care and use program is routinely reviewed by an institutional animal care and use committee and the U.S. Navy Bureau of Medicine. The animal use and care protocol for Navy dolphins in support of this study was approved by NIWC Pacific’s Institutional Animal Care and Use Committee (No. 134-2019) and the U.S. Navy Bureau of Medicine (NRD-1198).

### 2.2 Anesthesia

Food was withheld from dolphins for at least 18 h prior to each anesthetic procedure. Two dolphins, however, received oral diazepam sedation (5 mg tablets, Teva Pharmaceuticals United States, Inc., Parsippany, NJ 07054, United States; 0.08–0.30 mg/kg PO) in one capelin (*Mallotus villosus*) 1–2 h prior to injectable pre-medication. All dolphins were pre-medicated with a combined midazolam (West-Ward, Eatontown, NJ 07724, USA; 0.08–0.1 mg/kg IM) and meperidine (Hikma Pharmaceuticals, Berkeley Heights, NJ 07922 United States; 0.1–0.2 mg/kg IM) injection. A 5 Fr, 10 cm catheter (Micro-introducer Kit, Innovative Veterinary Medicine, Inc., Ponte Vedra, FL 32081, United States) was aseptically placed, using ultrasound guidance, into a lateral caudal subcutaneous vein (49). Immediately prior to and during induction of anesthesia, a 5 mL/kg IV bolus of lactated Ringer’s solution (Baxter Healthcare Corporation, Deerfield, IL 60015, United States) was administered. General anesthesia was induced with midazolam (0.02 mg/kg IV) and propofol (Hospira, Inc., Lake Forest, IL 60045 USA; 2–4 mg/kg IV) to allow for orotracheal intubation (16–18 mm internal diameter, cuffed silicone tracheal tube). Anesthesia was maintained in sternally-recumbent dolphins using sevoflurane (Baxter Healthcare Corporation, Deerfield, IL 60015, United States) and 100% oxygen on a circle breathing system. Anesthetic gas delivery was adjusted to maintain 1.8%–2.0% end-tidal sevoflurane concentration (F_Esevo_). Intravenous lactated Ringer’s solution infusion was maintained at 5 mL/kg/h throughout the anesthetic procedure. All dolphins were temporarily placed into lateral, oblique position to allow placement of a 4 Fr, 10 cm palmar median artery catheter (Innovative Veterinary Medicine, Inc., FL, USA) for continuous direct blood pressure measurement, arterial blood gas sampling, and cardiac output determination via lithium dilution (LiDCO Unity; LiDCO, London, N17 0QJ, United Kingdom) (50).

Additional anesthesia and analgesia were provided, when clinically indicated, in the form of regional or local nerve blocks (lidocaine, 2% inj, Hospira, Inc.; bupivacaine, 0.5% inj, Hospira, Inc.) and/or midazolam (0.02 mg/kg) and/or meperidine (0.1–0.2 mg/kg) IV boluses. In one dolphin, neuromuscular blockade (atracurium besylate, Hospira, Inc., 0.1 mg/kg IV, PRN q20 mins) was used to provide muscle relaxation. Table 3 provides a summary of anesthetic protocols used on individual animals.

**Table 3:** Summary of balanced anesthetic protocols for bottlenose dolphins enrolled in the study.

	Pre-medication			Induction		Maintenance				Reversal		
Animal ID	Diaz (mg/kg)	Midaz (mg/kg)	Meper (mg/kg)	Midaz (mg/kg)	Propofol (mg/kg)	Sevo (FE%)	Midaz (mg/kg)	Meper (mg/kg)	Atrac (mg/kg)	Fluma (mg/kg)	Nalox (mg/kg)	Naltrex (mg/kg)
Tt-1a^*^	-	0.08	0.10	0.02	2.30	1.8–2.0	-	-	-	0.02	0.02	-
Tt-2	-	0.08	0.10	0.02	2.00	1.8–2.0	-	-	-	0.02	0.01	-
Tt-3	0.3	0.10	0.20	0.02	3.43	1.8–2.0	-	-	-	0.03	0.02	0.05
Tt-1b*	-	0.10	0.20	0.02	4.89	1.8–2.0	-	-	-	0.02	0.04	-
Tt-5	0.08	0.10	0.10	0.02	1.73	1.8–2.0	0.04	0.1	-	0.02	-	0.05
Tt-6	-	0.08	0.10	0.02	2.00	1.8–2.0	0.03	0.1	-	0.02	0.01	-
Tt-7	-	0.12	0.10	0.02	2.91	1.8–2.0	-	-	-	0.04	-	0.10
Tt-8	-	0.08	0.10	0.02	3.02	1.8–2.0	0.06	0.1	-	0.02	-	0.15
Tt-9	-	0.10	0.10	0.02	2.42	1.8–2.0	-	-	-	0.02	0.02	0.15
Tt-10	-	0.08	0.10	0.02	2.25	1.8–2.0	0.12	-	0.9	0.05	0.02	0.20
Mean doses	0.19	0.09	0.12	0.02	2.70	1.8–2.0	0.06	0.1	0.9	0.03	0.02	0.12

Overall dose means shown on the bottom row. Diaz, diazepam; Midaz, midazolam; Meper, meperidine; Sevo, sevoflurane; Atrac, atracurium; Fluma, flumazenil; Nalox, naloxone; Naltrex, naltrexone. ^*^Denotes same female dolphin anesthetized on two separate occasions.

### 2.3 Study design and criteria

The two modes of ventilation were applied to each dolphin in a cross-over design. CMV was applied using a commercially available large animal anesthesia machine with a descending bellows ventilator (Surgivet LDS3000 with DHV1000; Smiths Medical ASD Inc., OH, United States). AAV was applied using a custom-built ventilator (DolVent; Innovative Veterinary Medicine, LLC., FL, United States) adapted to drive the same commercially available bellows assembly. Ventilation modes were applied based on previously published implementation strategies and estimates of tidal volumes for dolphins—maximum 20 mL/kg tidal volume (V_T_) and an arterial carbon dioxide tension (PaCO_2_) within 50–55 mmHg (31, 51). Mean arterial blood pressure (MAP) was maintained between 65 and 80 mmHg using ephedrine (Akorn, Inc., Lake Forest, IL 60045, United States; 0.05 mg/kg IV) followed by a constant rate infusion (CRI) of dobutamine (Hospira, Inc., 0.1–2 mcg/kg/min IV). Phenylephrine (Avadel Legacy Pharmaceuticals, LLC, Chesterfield, MO 63005, United States; 0.01–1 mcg/kg/min IV) CRI was supplemented to manage hypotension when the dobutamine CRI was insufficient. Table 4 provides a summary of hemodynamic support used for managing blood pressure in individual dolphins.

**Table 4:** Summary of hemodynamic support for bottlenose dolphins enrolled in the study.

Animal ID	Sex	Age	Weight (kgs)	Ephedrine (mg/kg)	Dobutamine (mcg/kg)	Phenylephrine (mcg/kg)
Tt-1a^*^	F^*^	37	187	-	-	-
Tt-2	M	29	191	0.05	-	-
Tt-3	M	33	254	0.10	75.63	32.52
Tt-1b^*^	F^*^	38	188	0.10	30.85	-
Tt-5	M	42	260	0.05	17.92	3.27
Tt-6	F	16	141	0.05	13.05	5.67
Tt-7	M	40	292	0.10	43.84	74.66
Tt-8	F	40	199	0.10	28.14	16.58
Tt-9	M	10	186	0.05	1.41	-
Tt-10	M	37	191	0.10	54.83	88.06
Mean	-	32	209	0.08	33.13	36.79

Total doses provided over the entire anesthetic period are shown. Ephedrine was provided as individual IV injections. Dobutamine and phenylephrine provided as IV constant rate infusions. Overall dose means shown. ^*^Denotes same female dolphin anesthetized on two separate occasions.

**Figure 2:**
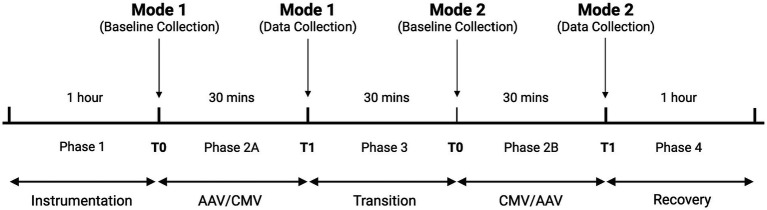
Cross-over study design to compare two ventilation modes in bottlenose dolphins. Order of ventilation mode was randomly determined. Times in each phase are approximate. Figure created using Biorender.com.

Four phases occurred in all dolphin procedures: Phase 1—instrumentation; Phase 2A—first ventilation mode; Phase 3—transition; Phase 2B—alternate ventilation mode; Phase 4—recovery (Figure 2). During Phase 2A and 2B, data were collected at baseline (T0) and at 30 min (T1) after baseline. A 30-min transition interval (Phase 3) was allotted between Phases 2A and 2B for switching ventilation modes and adjusting ventilatory settings to meet study criteria. Surgical and medical procedures occurred throughout the study period but were temporarily halted to allow for steady-state data collection and physiologic measurements at each T0 and T1. During Phase 4, reversal agents flumazenil (Fresenius Kabi, Lake Zurich, IL 60047, United States; 1:13 IV) and naloxone (International Medication Systems Limited, El Monte, CA 91733, United States; 10 mcg/kg IV) were administered once F_Esevo_ consistently measured less than 0.8% (Carescape B650; GE Healthcare, Chicago, IL 60661, United States). All dolphins recovered from general anesthesia without complications.

### 2.4 Monitoring and data collection

A multiparameter monitor (Carescape B650, GE Healthcare) was used for monitoring base-apex electrocardiogram, pulse oximetry, side-stream airway gases (F_Esevo_ and ETCO_2_) and MAP. Calibration of the airway gas module (755571-HEL, GE Healthcare) according to manufacturer specifications was performed prior to each anesthetic procedure.

A calibrated, pitot tube differential pressure spirometer (DolVent EUI; Innovative Veterinary Medicine, Inc., FL, United States) was placed between the breathing system and the tracheal tube for measurement of respiratory variables. Spirometer accuracy was assessed prior to each anesthetic procedure using a 7 L calibrated syringe (Model 4,900; Hans Rudolph Inc., KS, United States) to ensure measured volumes were within +/− 100 mL of delivered volumes (<2%). Measured and calculated variables common to both ventilation modes included V_T_, respiratory rate (*f*_R_), mean airway pressure (P_aw_), and total respiratory system dynamic compliance (C_RS_). CMV specific variables included inspiratory-to-expiratory ratio (I:E), peak inspiratory pressure (PIP), and end-expiratory pressure (EEP). AAV specific variables included P_high_, P_low_, T_high_, and T_low_. For CMV and AAV, C_RS_ was computed as V_T_/(PIP − EEP) and V_T_/(P_high_ − P_low_), respectively.

To avoid the need for right heart catheterization in the study dolphins, cardiac output (CO) was measured via lithium dilution (LiDCO Unity) administered in a peripheral vein (31, 50, 52). A sterile lithium chloride solution (lithium chloride, powder, Sigma-Aldrich Inc., St. Louis, MO 63103, United States; sterile water, inj, APP Pharmaceuticals, LLC, Schaumburg, IL 60173, United States) was prepared prior to each anesthetic procedure and a 0.01 mmol/kg IV bolus given for each cardiac output determination.

At each data collection time point, cardiopulmonary data were recorded, systemic arterial (pH_a_, PaO_2_, PaCO_2_, hemoglobin, and oxyhemoglobin saturation) and venous (pHv, PvO_2_, PvCO_2_, oxyhemoglobin saturation) blood samples were simultaneously collected (safePICO Aspirator; Radiometer Medical ApS, Copenhagen 2700, Denmark) from the lateral caudal subcutaneous vein and the palmar median artery for immediate analysis (ABL90 Flex, Radiometer Medical ApS), and CO was measured.

### 2.5 Calculated data

Data calculated using standard formulae included stroke volume (SV = CO/HR), alveolar dead space as a fraction of alveolar ventilation [V_Dalv_/V_Talv_ = (PaCO_2_ − ETCO_2_)/PaCO_2_], alveolar-arterial oxygen tension difference [P_A_O_2_ – PaO_2_, where P_A_O_2_ = (P_B_ – P_H20_) – P_A_CO_2_/R]; PB is barometric pressure, P_H20_ is the saturated vapor pressure of water at 37°C, P_A_CO_2_ is alveolar PCO_2_ tension (approximated by PaCO_2_), R is the respiratory quotient and assumed equal to 0.8, systemic vascular resistance (SVR = MAP/CO), arterial blood oxygen content (CaO_2_ = 1.36 × [Hb] × SaO_2_ + 0.003 × PaO_2_), and oxygen delivery (DO_2_ = CaO_2_ × CO). CO, SV, DO_2,_ V_T_, and C_RS_ were indexed to body mass and denoted by an appended I.

### 2.6 Statistical analysis

Statistical modeling of the cardiopulmonary data utilized repeated measures three-way ANOVA with main factors mode of ventilation, time point, and order (SAS Version 9.4; SAS Institute, NC, United States). The full model, with specified covariance structure, was fit and assumptions were checked, including residual diagnostics and the assumption of normality. To examine if order of ventilation mode was statistically significant, a re-parameterized two-way ANOVA model with two main factors (order, 2 levels, and time point-by-mode, 4 levels) was used to facilitate contrast estimation. When interactions were not significant, main effects were interpreted using the overall F-statistic and *p*-value, followed by *post hoc* mean comparisons and contrast estimation using Tukey’ simultaneous inference *p*-value adjustment. Statistical significance was set at *p* < 0.05.

## 3 Results

Comprehensive health evaluations indicated the physical health statuses of all dolphins were appropriate for general anesthesia and participation in the study. Descriptive statistics for variables specific to each mechanical ventilation mode are provided in Table 5. Three factors included in the final statistical model were order, time point, and ventilation mode. No significant differences were found within a mode between time points for all variables.

**Table 5:** Descriptive statistics of variables recorded during conventional mechanical ventilation (CMV) and apneustic anesthesia ventilation (AAV) modes in nine sternally recumbent anesthetized dolphins (10 anesthetic procedures).

Ventilation mode	Variable	Mean ± SE
CMV	PIP (cmH_2_O)	31.4 ± 0.55
	I:E	0.25 ± 0.01
	EEP (cmH_2_0)	2.1 ± 0.07
AAV	P_HIGH_ (cmH_2_O)	26.5 ± 0.55
	P_LOW_ (cmH_2_O)	2 ± 0.19
	T_HIGH_ (s)	19.73 ± 1.08
	T_LOW_ (s)	1.53 ± 0.07

Data presented as mean of all time points ± standard error of the mean. PIP, peak inspiratory pressure; I:E, inspiratory-to-expiratory ratio; EEP, end expiratory pressure; P_HIGH_, high pressure; P_LOW_, low pressure; T_HIGH_, time at P_HIGH_; T_LOW_, time at P_LOW_.

An effect of mode of ventilation at all time points was found for *f*_R,_ PaO_2,_ PaCO_2,_ pHa, ETCO_2_, venous oxygen tension (PvO_2_), V_Dalv_/V_Talv_, P_A_O_2_-PaO_2,_ mean Paw, C_RS_I, and systolic arterial pressure (SysAP; Tables 6, 7). Respiratory rate (*f*_R_; *p* < 0.0001) and PaCO_2_ (*p* = 0.022) were lower and ETCO_2_ (*p* < 0.0001) was higher in AAV compared to CMV. pHa was higher (i.e., closer to normal, physiologic pH) on AAV compared to CMV (*p* = 0.0121). PaO_2_ and PvO_2_ were significantly higher on AAV compared to CMV (*p* < 0.0001 and *p* = 0.039, respectively). Mean airway pressure (Paw) and respiratory system dynamic compliance index (C_RS_I) were significantly higher on AAV (*p* < 0.0001). Alveolar dead space ventilation (V_Dalv_/V_Talv_) and alveolar-arterial oxygen tension difference (P_A_O_2_-PaO_2_) were lower on AAV compared to CMV (*p* < 0.0001). While SysAP was significantly lower on AAV (*p* = 0.0021), MAP and diastolic arterial pressure (DiasAP) were not statistically significant at *p* = 0.0504 and 0.0610, respectively. The statistically significant effects of ETCO_2_ were not considered clinically significant, as the study criteria aimed to maintain a narrow range of PaCO_2_ by titrating ETCO_2_ using respiratory rate. Additionally, inherent differences in *f*_R_ between the two ventilation modes likely affected accurate comparison of ETCO_2._ No statistically significant differences between modes were found for HR, MAP, DiasAP, CI, pHv, venous carbon dioxide tension (PvCO_2_), pulse oximetry (SpO_2_), SV index (SVI), and SVR.

**Table 6:** Measured and calculated respiratory variables in nine sternally recumbent anesthetized dolphins (10 anesthetic procedures) ventilated with either apneustic anesthesia ventilation (AAV) or conventional mechanical ventilation (CMV).

Variable	AAV mode	CMV mode	Mode effect	Order effect	
				AAV	CMV
*f*_R_ (breaths min^−1^)	3 ± 0.2	5.2 ± 0.2	<0.0001	0.1490	0.8264
V_T_I (mL kg^−1^)	18.8 ± 0.6	19.4 ± 0.2	0.2773	0.0336	0.1413
PaO_2_ (mmHg)	503.1 ± 17	276 ± 27	<0.0001	<0.0001	<0.0001
PaCO_2_ (mmHg)	49.7 ± 0.9	52.2 ± 1	0.022	0.2198	0.3498
pHa	7.29 ± 0.01	7.26 ± 0.01	0.0121	0.2086	0.2602
ETCO_2_ (mmHg)	50.6 ± 1.2	40.8 ± 0.9	<0.0001	0.0007	0.0406
V_Dalv_/V_Talv_	−0.02 ± 0.01	0.22 ± 0.02	<0.0001	0.0346	0.8691
P_A_O_2_-PaO_2_ (mmHg)	148.1 ± 16	372.5 ± 27	<0.0001	<0.0001	<0.0001
Mean Paw (cmH_2_O)	23.2 ± 0.5	7.2 ± 0.2	<0.0001	0.5600	0.9620
C_RS_I (mL cmH_2_O^−1^ kg^−1^)	0.77 ± 0.02	0.67 ± 0.01	<0.0001	0.0129	0.3570

No significant differences were noted between time points within each mode, therefore, data presented as mean of all time points within a ventilation mode ± standard error of the mean. Overall effects (*p*-values) of ventilation mode and order of mode application are shown. *f*_R_, respiratory (CMV) or release (AAV) rate; V_T_I, tidal volume index; PaO_2_, arterial oxygen tension; SaO_2_, arterial hemoglobin saturation; PaCO_2_, arterial carbon dioxide tension; pHa, arterial pH; ETCO_2_, end-tidal carbon dioxide tension; V_Dalv_/V_Talv_, alveolar dead space as a fraction of alveolar ventilation; P_A_O_2_-PaO_2_, alveolar-arterial oxygen tension gradient; Mean Paw, mean airway pressure; C_RS_I, respiratory system dynamic compliance index.

**Table 7:** Measured and calculated cardiovascular variables in nine sternally recumbent anesthetized dolphins (10 anesthetic procedures) ventilated with either apneustic anesthesia ventilation (AAV) or conventional mechanical ventilation (CMV).

Variable	AAV mode	CMV mode	Mode effect	Order effect	
				AAV	CMV
HR (beats min^−1^)	94 ± 1.7	95 ± 1.8	0.2278	0.0031	0.0145
MAP (mmHg)	80.8 ± 2.9	86 ± 2.6	0.0504	0.0041	0.1579
SysAP (mmHg)	114.1 ± 2.5	123.4 ± 2.1	0.0021	0.1336	0.2777
DiasAP (mmHg)	65.8 ± 2.9	70.5 ± 2.6	0.0610	0.0045	0.0836
CI (mL min^−1^ kg^−1^)	140.8 ± 6.9	149.2 ± 6.6	0.0692	0.1890	0.1128
SVI (mL beat^−1^ kg^−1^)	1.48 ± 0.06	1.57 ± 0.07	0.0767	0.0624	0.0313
SVR (dyne s cm^−5^)	233 ± 12	236 ± 14	0.6973	<0.0001	0.0004
CaO_2_ (mL dL^−1^)	20.87 ± 0.40	20.33 ± 0.43	0.0002	<0.0001	<0.0001
DO_2_I (mL min^−1^ kg^−1^)	29.6 ± 1.8	30.6 ± 1.7	0.2829	0.0237	0.0167

No significant differences were noted between time points within each mode, therefore, data presented as mean of all time points within a ventilation mode ± standard error of the mean. Overall effects (*p*-values) of ventilation mode and order of mode application are shown. HR, heart rate; MAP, mean arterial pressure; SysAP, systolic arterial pressure; DiasAP, diastolic arterial pressure; CI, cardiac index; SVI, stroke volume index; SVR, systemic vascular resistance; CaO_2_, arterial blood oxygen content; DO_2_I, oxygen delivery index.

An effect of order of ventilation mode was found for V_T_I_,_ PaO_2_, ETCO_2_, V_Dalv_/V_Talv_, P_A_O_2_-PaO_2_, C_RS_I, MAP, DiasAP (Tables 6, 7). When AAV was applied as the first ventilation mode (Phase 2A), V_T_I and P_a_O_2_ were significantly higher compared to when CMV was the first mode applied (*p* = 0.0335 and *p* < 0.0001, respectively). V_Dalv_/V_Talv_ and P_A_O_2_-PaO_2_ were significantly lower (*p* = 0.035 and *p* < 0.0001, respectively) and C_RS_I was significantly higher when AAV was applied as the first mode (*p* = 0.0129). When AAV was the first mode used, MAP and DiasAP were significantly lower than when CMV was applied first (*p* = 0.0041 and 0.0045, respectively). Finally, SVI was significantly higher when CMV was applied first (*p* = 0.0313).

## 4 Discussion

In this study, we assessed the cardiopulmonary effects of two distinct modes of mechanical ventilation on anesthetized bottlenose dolphins to determine if a novel mode, AAV, may provide enhanced cardiopulmonary performance compared to CMV.

Central to this study are the significant differences in clinically relevant pulmonary variables when comparing the two ventilation modes. Despite provision of ~98% inhaled oxygen and targeting a PaCO_2_ range between 50 and 55 mmHg via continuous ETCO_2_ measurements during both ventilation modes, AAV exhibited significantly higher PaO_2_, significantly less alveolar dead space ventilation (V_Dalv_/V_Talv_), and lower alveolar-arterial oxygen tension differences (P_A_O_2_-PaO_2_) than CMV. This would imply improved pulmonary function during AAV and less ventilation-perfusion scatter, whereby most of the dolphin’s lung volume would be adequately perfused and able to effectively participate in gas exchange. Additionally, respiratory system compliance index (C_RS_I) was significantly higher on AAV, supporting larger tidal volumes over smaller changes in airway pressure, thus improving gas exchange as evidenced from the lower alveolar dead space ventilation (i.e., the proportion of alveolar ventilation not participating in gas exchange).

Improved assessment of ventilation-perfusion matching in the study dolphins would require venous admixture calculation with pulmonary arterial blood and measurement of pulmonary arterial and pulmonary arterial occlusion pressures, increasing the risk of adverse events and adding the complexity of pulmonary arterial vascular access in this species (53, 54). For this reason, central cannulation was not performed. Furthermore, current methods of estimating alveolar dead space in ventilated patients, as performed in this study, are based on end-tidal CO_2_ concentrations as approximations of mean alveolar CO_2_ in healthy subjects, and therefore, less accurate measurements of Bohr dead space (55). While all dolphins in this study received comprehensive health evaluations, presence of subclinical, or asymptomatic, pulmonary disease cannot be completely excluded.

The effects of positive pressure ventilation in either mode of mechanical ventilation would be expected to increase intrathoracic pressures and contribute to reduced cardiac preload, cardiac output, blood pressure, and subsequently reduced oxygen delivery to tissues. An important determinant of the cardiovascular effects of positive pressure ventilation is mean airway pressure (mean Paw), whereby increasing airway pressures can contribute to decreasing preload. During implementation of AAV in the study dolphins, mean Paw was significantly higher due to the proportion of time spent at P_HIGH_ (approximately 16 mmHg higher than CMV Paw). However, overall, P_HIGH_ on AAV was lower than PIP on CMV. In a clinical setting, P_HIGH_ and P_LOW_ should be applied to optimize dynamic compliance of the respiratory system to prevent progression of atelectasis. The cardiovascular effects of the higher mean Paw were mostly observed when AAV was the first mode implemented in the study dolphins. For example, when AAV was the first mode of mechanical ventilation, MAP and SVR were significantly lower. AAV displayed lower SV while CMV showed higher SVI with no significant differences in the other cardiovascular variables. However, these findings are likely attributable to the low number of animals enrolled in the study or to the pre-established criteria of a narrow MAP maintenance range, most often requiring the use of inotropic and vasopressive agents to modulate cardiovascular responses. Additionally, the intentional hypercapnia component of the current study (50–55 mmHg) may have contributed to physiologic advantages, such as increased circulating catecholamine concentrations, which may support cardiovascular function and may have contributed to non-significance in most of the cardiovascular variables (56). Overall, from a clinical perspective, AAV did not appear to negatively affect the hemodynamic status of the study dolphins.

While this study provides evidence of improved pulmonary function in dolphins ventilated using the AAV strategy, a comprehensive understanding of normal, in-water dolphin physiology is critical to the understanding of the overall influences of general anesthesia on dolphin cardiopulmonary physiology. Having evolved and adapted to aquatic environments, dolphin circulation, perfusion, and efficiency of gas exchange are likely altered by the removal of minimal to neutral buoyancy for an anesthetized procedure. In one study with 4 out-of-water dolphins anesthetized with a nitrous oxide gas mixture, the neuromuscular blocking agent succinyldicholine, and intraperitoneal or intravenous thiopental and mechanically-ventilated, SVI and CI directly measured between 0.4–0.8 mL/kg and 47–105 mL/min/kg, respectively (57). Directly measured MAP ranged between 122 and 142 mmHg (systolic: 142–160 mmHg; diastolic: 111–130 mmHg). In another study using halothane gas and 10 mg/kg intravenous thiopental, MAP in the study dolphins averaged 115 mmHg, where normal MAP was considered 120–140 mmHg (37). Dolphins in these studies were ventilated using a modified large animal ventilator with a ventilation strategy termed apneustic plateau ventilation, whereby rapid inflation was followed by an inspiratory breath hold and rapid pressure release (37, 58, 59). This mode did not include end expiratory pressure control methods and may have contributed to progressive atelectasis in the study dolphins.

In other studies, using non-invasive methods on non-anesthetized in-water dolphins, average calculated SVI and CI were 0.8 mL/kg and 32.2 mL/min/kg, respectively (60). The dolphins in the current study had SVI measuring 1.5 ± 0.1 mL/kg and CI measurements between 139 and 154 mL/min/kg, with no significant differences observed between the two ventilation modes. Average MAP was between 80.8 and 86 mmHg throughout all phases of the study. Although limited data is available for comparison, the increased SVI and CI and lower MAP measured in anesthetized out-of-water dolphins compared to non-anesthetized in-water dolphins could be attributable to differences in measurement techniques or anesthesia-associated modulation of normal circulation (i.e., post-intubation hypotension, hyperoxia, baroreceptor reflex, differences in mechanical ventilation strategies, and/or anesthetic agents). While these measurements were acquired under general anesthesia and out-of-water conditions, it is difficult to discern the effects of removal from a neutrally buoyant condition from the effects of anesthesia on the measured cardiovascular indices. The comparably lower arterial blood pressure readings in the current study, however, may elucidate an anesthesia-associated hypotensive state of current protocols when compared to anesthetized dolphins from earlier studies, where MAP ranged between 115 and 142 mmHg (37, 57).

While this is the first controlled study comparing to two distinct modes of ventilation in dolphins, several study limitations may have influenced measured and calculated variables, including lack of age and sex matching in the study population, measurement errors, and large gaps in the pharmacokinetic and pharmacodynamic understanding of the anesthetic drugs used. Additionally, interpretation of the cardiovascular variables subjected to statistical analysis may have been confounded by the use of ephedrine boluses and dobutamine and phenylephrine constant rate infusions to maintain the study’s target MAP range of 65–80 mmHg.

## 5 Conclusion

General anesthesia of this completely aquatic mammal is constrained by several logistical and physiologic complexities, briefly described herein. This study aimed to evaluate one such physiologic complexity, the ventilation-induced cardiopulmonary responses of dolphins, using two distinct ventilation strategies, while attempting to control for variables that would inherently confound interpretation of these responses (i.e., targeted MAP range, pain-associated cardiovascular modulation). While both modes of ventilation provided adequate ventilation-perfusion matching and tissue oxygen delivery at 100% inspired oxygen, AAV resulted in a lower alveolar to arterial oxygen gradient, higher dynamic respiratory system compliance index, significantly higher mean airway pressures, and less alveolar dead space compared to CMV. Additionally, AAV resulted in significantly decreased indices of some parameters of cardiovascular performance (MAP and DiasAP) when implemented as the first mode of ventilation. However, regardless of order of implementation, there were no significant differences in cardiovascular variables between the modes.

Clinical application of mechanical ventilation in dolphins should, therefore, prioritize individual patient needs when possible. Additional complexities of dolphin anesthesia and physiology to consider and mitigate include modulation of core body temperature, patient padding to reduce myopathic and neuropathic morbidities, physiologic effects of removal from neutral buoyancy, and logistics of anesthetic emergence and surgical wound healing. Future studies should, therefore, assess the effects of the out-of-water condition on dolphin circulation and pulmonary function, to include establishment of normal in-water mean arterial pressures, in order to better inform veterinarians on physiologically appropriate anesthetic approaches.

## Data availability statement

The original contributions presented in the study are included in the article, further inquiries can be directed to the corresponding author.

## Ethics statement

The animal study was approved by Naval Information Warfare Center Pacific, Institutional Animal Care and Use Committee. The study was conducted in accordance with institutional requirements (No. 134-2019) and the U.S. Navy Bureau of Medicine (NRD-1198).

## Author contributions

CL-B: Conceptualization, Data curation, Formal analysis, Funding acquisition, Investigation, Methodology, Project administration, Resources, Supervision, Validation, Visualization, Writing – original draft, Writing – review & editing. AB: Conceptualization, Data curation, Formal analysis, Investigation, Methodology, Resources, Supervision, Validation, Visualization, Writing – review & editing. JD: Conceptualization, Formal analysis, Investigation, Methodology, Resources, Supervision, Visualization, Writing – review & editing. DH: Conceptualization, Data curation, Formal analysis, Investigation, Methodology, Validation, Visualization, Writing – review & editing. LT: Formal analysis, Methodology, Validation, Writing – review & editing. SR: Conceptualization, Funding acquisition, Methodology, Resources, Supervision, Validation, Visualization, Writing – review & editing. JB: Conceptualization, Data curation, Formal analysis, Funding acquisition, Investigation, Methodology, Project administration, Resources, Software, Supervision, Validation, Visualization, Writing – review & editing.

## In memoriam

The authors would like to acknowledge a co-author, SR, a remarkable scientist and the original dolphin anesthesiologist who, unfortunately, passed away in 2022 prior to completion of this manuscript. Thank you for entrusting us with this work. We dedicate this manuscript in your honor.
